# The Georgia Pharmaceutical Association

**Published:** 1877-05-20

**Authors:** 


THE Georgia Pharmaceutical Association, held their second annual meeting in Atlanta recently. It was numerously attended. The following officers were elected: President—R. H. Land, Augusta; First Vice President—E. W. Hunter, Louisville; Second Vice President—Roland B. Hall, Macon; Third Vice President—O. Butler, Savannah; Treasurer—John Ingalls, Macon; Secretary—W. A. Taylor, Atlanta.

